# Ensemble learning for integrative prediction of genetic values with genomic variants

**DOI:** 10.1186/s12859-024-05720-x

**Published:** 2024-03-21

**Authors:** Lin-Lin Gu, Run-Qing Yang, Zhi-Yong Wang, Dan Jiang, Ming Fang

**Affiliations:** 1 Key Laboratory of Healthy Mariculture for the East China Sea, Ministry of Agriculture and Rural Affairs and Fisheries College, Jimei University, Xiamen, People’s Republic of China; 2 Research Center for Aquatic Biotechnology, Chinese Academy of Fishery Sciences, Beijing, People’s Republic of China; 3 Life Science College, Heilongjiang Bayi Agricultural University, Daqing, People’s Republic of China

**Keywords:** Genomic prediction, Genomic selection, Ensemble learning, Machine learning

## Abstract

**Background:**

Whole genome variants offer sufficient information for genetic prediction of human disease risk, and prediction of animal and plant breeding values. Many sophisticated statistical methods have been developed for enhancing the predictive ability. However, each method has its own advantages and disadvantages, so far, no one method can beat others.

**Results:**

We herein propose an Ensemble Learning method for Prediction of Genetic Values (ELPGV), which assembles predictions from several basic methods such as GBLUP, BayesA, BayesB and BayesCπ, to produce more accurate predictions. We validated ELPGV with a variety of well-known datasets and a serious of simulated datasets. All revealed that ELPGV was able to significantly enhance the predictive ability than any basic methods, for instance, the comparison *p*-value of ELPGV over basic methods were varied from 4.853E−118 to 9.640E−20 for WTCCC dataset.

**Conclusions:**

ELPGV is able to integrate the merit of each method together to produce significantly higher predictive ability than any basic methods and it is simple to implement, fast to run, without using genotype data. is promising for wide application in genetic predictions.

## Background

Genome-wide distributed variants provide sufficient information for prediction of genetic value. In human studies, genetic value prediction is usually applied for prediction of complex traits such as disease risk and human height [1, 2]. In plants and animals, genetic prediction is important for genetic selection [3, 4]. So far, a variety of statistical analysis methods have been used to predict genetic values. Genomic best linear unbiased estimates (GBLUP) are the most common method, which uses genome-wide molecular markers to construct a kinship matrix between individuals and then uses BLUP techniques to predict individual genetic values [5]. Bayesian method is another popular method, which mainly includes BayesA, BayesB, BayesCπ and BayesLASSO, etc. [6–8]. These methods use Monte Carlo Markov chain techniques to estimate parameters. The main difference among them is the assignment of hyperparameters for variables, and each method has its own advantages and disadvantages. BayesA is mainly applicable for the traits controlled by genes with multiple tiny effects, whereas BayesB and BayesCπ are suitable for the traits controlled by a small number of main effect genes. In human disease risk prediction, “Clumping + Thresholding” (C + T) method has been developed and applied [9–12]. C + T method first identifies a set of markers with predictive power, and then uses these markers to predict disease risk by logistic regression [1, 13, 14], which is suitable for the disease controlled by several main effect genes. Although several methods exist, each has its own limitations, so far, there is no one method that always outperforms others.

Ensemble learning is a machine learning method, which integrates the predictions from multiple methods to obtain a new prediction through supervised or unsupervised learning methods [15]. As early as 20 years ago, it was found that ensemble learning can reduce generalization error [16] and ensemble methods that combine the output of multiple methods have been shown to achieve better generalizability than a single method [17]. So far, ensemble learning has independently made a substantial impact on the field of bioinformatics through their widespread applications [18]. One example is in predicting localization of long non-coding RNAs, where multiple sub-networks were used to integrate distinct feature sets to maximize method performance [19]. In another work, a CNN/RNN (Convolutional Neural Networks/Recurrent Neural Network) ensemble was used to integrate features and raw sequence data to predict different types of translation initiation sites [20], overcoming the generalizability issue of traditional methods that can only predict a specific type of translational initiation sites. Moreover, the stability and reproducibility offered by ensemble methods such as in feature selection are also making a substantial impact in biomarker discovery [21, 22]. To our best knowledge, the remarkable flexibility and adaptability characters of ensemble learning has led to the proliferation of their application in bioinformatics research [23].

We herein propose an ensemble learning method for Prediction of Genetic Values (ELPGV). ELPGV trains several different basic methods, such as GBLUP, BayesA, BayesB and BayesCπ, to produce more accurate prediction. The core of ELPGV uses the hybrids of differential evolution [24] and particle swarm optimization [25] to train the weight, by which the predictions of basic methods are weighted averaged to generate new prediction. A variety of dataset including WTCCC (Wellcome Trust Case Control Consortium), IBDGC (International Inflammatory Bowel Disease Genetics Consortium), cattle, wheat and computational simulations are employed to validate ELPGV.

## Materials and methods

### Basic methods

The prediction is based on a linear method according to Eq. (1):

1 $${\varvec{y}} = \user2{X\alpha } + \user2{Z\beta } + {\varvec{e}}$$

where ${\varvec{y}}$ is the phenotypes; ${\varvec{X}}$ is design matrix for fixed effects; $\boldsymbol{\alpha }$ is the fixed effect; ${\varvec{Z}}$ is genotypes of variants, coding with “0”, “1” and “2” for genotypes “AA”, “Aa” and “aa” respectively, or genotype dosages of SNPs; ${\varvec{\beta}}$ is the SNP effects; and ${\varvec{e}}$ is the residual errors, assumed to follow normal distribution,${\varvec{e}}\sim N\left(0,{\varvec{I}}{\sigma }_{e}^{2}\right)$, where ${\varvec{I}}$ is a vector of identity matrix and ${\sigma }_{e}^{2}$ is the residual variance.

In this study, four basic methods are used for genetic value predictions, BayesA, BayesB, BayesCπ and GBLUP. In BayesA, all SNPs are assumed to contribute to genetic variation, and the variance of the SNP effect is assumed to follow inverse chi-square distribution; BayesB and BayesCπ assumes a small fraction ($\pi$) of SNPs have non-zero effects [6, 8], where $\pi$ is set as 0.1 in BayesB [26]. The Bayesian methods are implemented with the function “BGLR” in the R package “BGLR” [27]. In the GBLUP, the variances of all SNP effects are assumed to be equal, and then the genetic values are estimated with mixed model equation through kinship matrix constructed with SNPs [5]. The GBLUP is implemented using the function “emmreml” in the R package “EMMREML” [28].

### ELPGV model construction

**Fig. 1:**
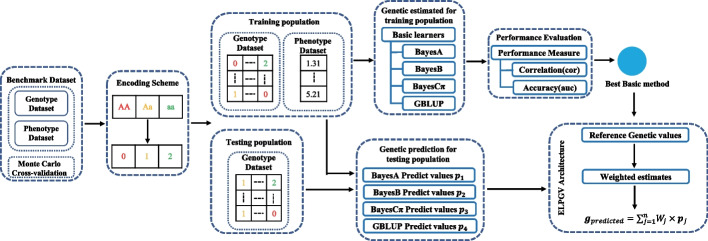
Schematic diagram of the study methodology

The ELPGV framework comprises two components, weight training and weighted prediction. First, it trains basic methods to get predictions; then, it trains the weight of basic methods with machine learning; finally, it generates new predictions by the weighted average of the predictions of basic methods. The schematic diagram of the study methodology is given in Fig. 1.

Suppose $n$ basic methods are investigated, the prediction of ELPGV can be expressed as Eq. (2), where, ${{\varvec{p}}}_{j}$ is the predicted values of the $j{\text{th}}$ basic method, which is easily obtained from each basic method, and ${W}_{j}$ is the weight of the $j{\text{th}}$ basic method, respectively.

2 $${\varvec{g}}_{predicted} = \mathop \sum \limits_{j = 1}^{n} W_{j} \times {\varvec{p}}_{j}$$

To train the weight ***W***, a fitness function is defined as the correlation coefficient between the predicted values ${{\varvec{g}}}_{predicted}$ and observed values ${{\varvec{y}}}_{observed}$ (Eq. 3), ${{\varvec{g}}}_{predicted}$ is the predicted values of ELPGV based on Eq. (2).

3 $$f\left( {\varvec{W}} \right) = \frac{{\sum \left( {{\varvec{y}}_{observed} - \overline{\user2{y}}_{observed} } \right)\left( {{\varvec{g}}_{predicted} - \overline{\user2{g}}_{predicted} } \right)}}{{\sqrt {\sum \left( {{\varvec{y}}_{observed} - \overline{\user2{y}}_{observed} } \right)^{2} } \sqrt {\sum \left( {{\varvec{g}}_{predicted} - \overline{\user2{g}}_{predicted} } \right)^{2} } }}$$

For testing population, phenotype ${{\varvec{y}}}_{observed}$ is unknown, we therefore introduce reference genetic values to replace the unknown phenotypic values in Eq. (3). The genetic predictions with the best fitness among basic methods was taken as the reference genetic values.

ELPGV uses a mixture of differential evolution (DE) algorithm and particle swarm optimization (PSO) algorithm to estimate the weight ***W***, which includes initialize, mutation, crossover and selection steps.

*Step 1. Initialization*: ELPGV randomly initializes the weights ${{\varvec{W}}}_{i\cdot }$(${W}_{i,1},\dots ,{W}_{i,j}$) and the optimization velocities ${{\varvec{V}}}_{i\cdot }$(${V}_{i,1},\dots ,{V}_{i,j}$), for $i=1,\dots ,m$ and $j=1,\dots ,n$, where *m* is the number of particles or the number of candidate weight; and *n* is the number of basic methods. The weight is initialized with Eq. (4) and the optimization velocity is initialized with Eq. (5).

4 $$W_{i,j} = rand\left( {W_{min} , W_{max} } \right)$$

5 $$V_{i,j} = rand\left( {V_{min} ,V_{max} } \right)$$

First, the $m$ group weights are replaced into Eq. (2) to obtain the ELPGV predictions of $m$ groups, respectively; then the predictions are replaced into Eq. (3) to assess the corresponding fitness for each group. We then define the optimal weight ${\varvec{W}}(0)$ as the best fitness one in all group weights,

6 $${\varvec{W}}\left( 0 \right) = argmax\left( {f\left( {{\varvec{W}}_{i\cdot} } \right)} \right)$$

*Step 2. Mutation*: In $t$ th iteration, Eq. (4) is replaced with Eqs. (7) and (8) for updating the weight of each group, respectively.

7 $${\varvec{P}}_{i\cdot} \left( t \right) = {\varvec{W}}_{i\cdot} \left( {t - 1} \right) + {\varvec{V}}_{i\cdot} \left( {t - 1} \right)$$

8 $${\varvec{H}}_{i\cdot} \left( t \right) = {\varvec{W}}_{k\cdot} \left( {t - 1} \right) + F \times \left( {{\varvec{W}}_{p\cdot} \left( {t - 1} \right) - {\varvec{W}}_{q\cdot} \left( {t - 1} \right)} \right)$$

where $F$ is scaling factor, controlling the effect of difference vector, the index $i\ne k\ne p$ $\ne q$.

*Step 3. Crossover*: The crossover operation switches the weight at current iteration $(t)$ and last iteration $\left(t-1\right)$ randomly with Eq. (9), where $CR$ is crossover probability and ${rand}_{i\cdot }\left(\mathrm{0,1}\right)$ is a random value between 0 and 1 of $i$ th group weight.

9 $${\varvec{U}}_{i\cdot} \left( t \right) = \left\{ {\begin{array}{*{20}c} {{\varvec{H}}_{i\cdot} \left( t \right)} & {rand_{i\cdot} \left( {0,1} \right) \le CR} \\ {{\varvec{W}}_{i\cdot} \left( {t - 1} \right)} & {else} \\ \end{array} } \right.$$

*Step 4. Selection*: Last, the all the group weights are updated with Eqs. (10) and (11).

10 $${\varvec{G}}_{i\cdot} \left( t \right) = \left\{ {\begin{array}{*{20}c} {{\varvec{U}}_{i\cdot} \left( t \right)} & {f\left( {{\varvec{U}}_{i\cdot} \left( t \right)} \right) \ge f\left( {{\varvec{P}}_{i\cdot} \left( t \right)} \right)} \\ {{\varvec{P}}_{i\cdot} \left( t \right)} & {else} \\ \end{array} } \right.$$

11 $${\varvec{W}}_{i\cdot} \left( t \right) = \left\{ {\begin{array}{*{20}c} {{\varvec{G}}_{i\cdot} \left( t \right)} & {f\left( {{\varvec{G}}_{i\cdot} \left( t \right)} \right) \ge f\left( {{\varvec{W}}_{i\cdot} \left( {t - 1} \right)} \right)} \\ {{\varvec{W}}_{i\cdot} \left( {t - 1} \right)} & {else} \\ \end{array} } \right.$$

After $t$ th iteration, each group weight has a velocity which are updated as Eq. (12), where $\varepsilon$ is inertia weight, ${c}_{1}$ and ${c}_{2}$ are accelerated factors.

12 $$\begin{aligned} & {\varvec{V}}_{i\cdot} \left( t \right) = \varepsilon *{\varvec{V}}_{i\cdot} \left( {t - 1} \right) + c_{1} *rand\left( {0,1} \right)*\left( {{\varvec{W}}_{i\cdot} \left( t \right) - {\varvec{W}}_{i\cdot} \left( {t - 1} \right)} \right) \\ & \quad + {\kern 1pt} c_{2} *rand\left( {0,1} \right)*\left( {{\varvec{W}}\left( {t - 1} \right) - {\varvec{W}}_{i\cdot} \left( {t - 1} \right)} \right) \\ \end{aligned}$$

At the same time, ELPGV updates the fitness with new weights at $t$ th updating with Eq. (3), the optimal weight can be expressed as Eq. (13) in $t$ th iteration.

13 $${\varvec{W}}\left( t \right) = argmax\left( {f\left( {{\varvec{W}}_{i\cdot} \left( t \right)} \right)} \right)$$

After the fitness meets a certain criterion, or the iterations reach the maximum number, ELPGV returns the optimal weights ${\varvec{W}}$ and the predictions with Eq. (2). To reduce sampling error and increase the estimate accurate of weights, the whole estimates are repeated for 100 times and the averaged weights are taken for ELPGV (Table 1).

**Table 1:** Lists the hyper parameters used in above equations

Parameters	Value
${W}_{min}$ *minimum weight*	0
${W}_{max}$ *maximum weight*	1
${V}_{min}$ *minimum update velocity*	− 0.01
${V}_{max}$ *maximum update velocity*	0.01
$m$ *the weight size*	20
$F$ *scaling factor*	0.5
$CR$ *crossover probability*	0.3
$\varepsilon$ *inertia weight*	1
${c}_{1}$ *accelerated factor 1*	2
${c}_{2}$ *accelerated factor 2*	2
$Max\_iterations$	25

### Monte Carlo cross-validation

Cross-validation was employed to evaluate the prediction performance of GS methods. The individuals of each dataset were first randomly divided into two parts with ratio 9:1, and they were taken as training set and testing set, respectively. The cross-validation was repeated 100 times. In the prediction, the phenotypes of individuals in testing set were masked, and the genetic values were predicted with training set; then the Pearson’s correlation coefficient between the predicted values and their true phenotypes were used to evaluate the predictive ability of each method.

### Paired-sample t-test

Because all the methods are compared with the same replicated dataset, we were able to compare ELPGV with other basic methods using paired-sample *t-*test, which is expressed as $t=\overline{d }/{s}_{\overline{d} }$, with degree of freedom $n-1$, where *n* is the times of cross validation and $d$ is the difference of the predictive ability between ELPGV and other methods.

### WTCCC dataset

The WTCCC dataset was accessed from the Wellcome Trust Case Control Consortium (WTCCC1, https://www.wtccc.org.uk/) [29], including 14,000 cases and 2,938 shared controls, all were genotyped for ~ 450,000 SNPs. Six diseases were investigated, including bipolar disorder (BD), coronary artery disease (CAD), hypertension (HT), rheumatoid arthritis (RA), type 1 diabetes (T1D), and type 2 diabetes (T2D). For this study, we removed SNPs using PLINK [30], with either minor allele frequency (MAF) < 0.01, or genotype call rate (CR) < 0.95, or *p*-value < 0.05 from Hardy–Weinberg equilibrium (HWE) test; then the SNPs were further pruned with PLINK [30] (r^2^ = 0.5) for reducing computational burden. The number of cases and the SNPs of each disease are shown in Table 2.

**Table 2:** Brief summary of the disease to WTCCC data sets

Disease	Case size	SNP size
BD	1868	373,369
CAD	1926	372,541
HT	1952	373,338
RA	1860	373,056
T1D	1963	372,964
T2D	1924	373,149

bipolar disorder (BD), coronary artery disease (CAD), hypertension (HT), rheumatoid arthritis (RA), type 1 diabetes (T1D) and type 2 diabetes (T2D)

### Inflammatory bowel disease (IBD) dataset

The inflammatory bowel disease dataset was accessed from the International IBD Genetics Consortium (IBDGC), including 20,155 Crohn disease (CD), 15,191 ulcerative colitis disease (UC) and 34,257 controls of European ancestry. In total, genotypes were called using optiCall for 192,402 autosomal variants before quality control. A total of 161,681 SNPs was available after removing the SNPs with MAF < 0.02 and *p*-value < 10e−5 from the HWE test. The missing genotypes were imputed with impute2 using 1000 genome as a reference. (For details, see refs. [31]. To reduce computation burden, we further pruned SNPs for linkage disequilibrium with threshold *r*^2^ = 0.5 using PLINK [30] and randomly sampled 1,000 individuals from Liege and Brussels batches.

### Cattle dataset

German Holstein genomic prediction population was further employed to validate ELPGV, which comprised 5024 bulls [32], and all were genotyped with the Illumina Bovine SNP50 Beadchip [33]. After removing the SNPs with HWE *p-*value < 10 − 4, CR < 0.95 and MAF < 0.01, a total of 42,551 SNPs remained for the downstream analysis. The estimated breeding values of three traits milk fat percentage (mfp), milk yield (my), and somatic cell score (scs) were available and used in this study.

### Wheat dataset

The wheat dataset was collected from CIMMYT’s Global Wheat Program, the grain yields (GY) of the 599 wheat inbred lines were recorded for four places [34, 35]. Each wheat line was genotyped with 1447 Diversity Array Technology (DArT) by Triticarte Pty. Ltd, which had two genotypes coded with “0” or “1”, to indicate its presence or absence, respectively, after filtering, 1279 markers were kept for analysis.

### Simulations

We took advantage of the genotypes of wheat datasets for simulation. A number of QTL were simulated with effects sampled from gamma distribution with scale parameter 1.66 and shape parameter 0.4; the residual errors were sampled from normal distribution with variance set according to the heritability. We performed two simulation experiments to investigate the performance of ELPGV: (1) simulation of different number of QTLs, 5 and 1,000, respectively; and (2) simulation of different heritabilities, 0.5 and 0.2, respectively. This led to four sets of experiments (QTL5 and h^2^ = 0.2, QTL5 and h^2^ = 0.5, QTL1000 and h^2^ = 0.2, QTL1000 and h^2^ = 0.5).

## Results

We used both real dataset and simulated dataset to validate the performance of ELPGV. In this study, four popular GS methods, GBLUP, BayesA, BayesB, and BayesCπ, were used for assembling with ELPGV, although ELPGV is able to assemble as many methods as possible. In addition, cross-validation was employed to evaluate the prediction performance of each method. Taken advantage of the fact that all the methods were compared with the same dataset, we used paired-sample *t*-test for significance comparison.

### WTCCC dataset

**Fig. 2:**
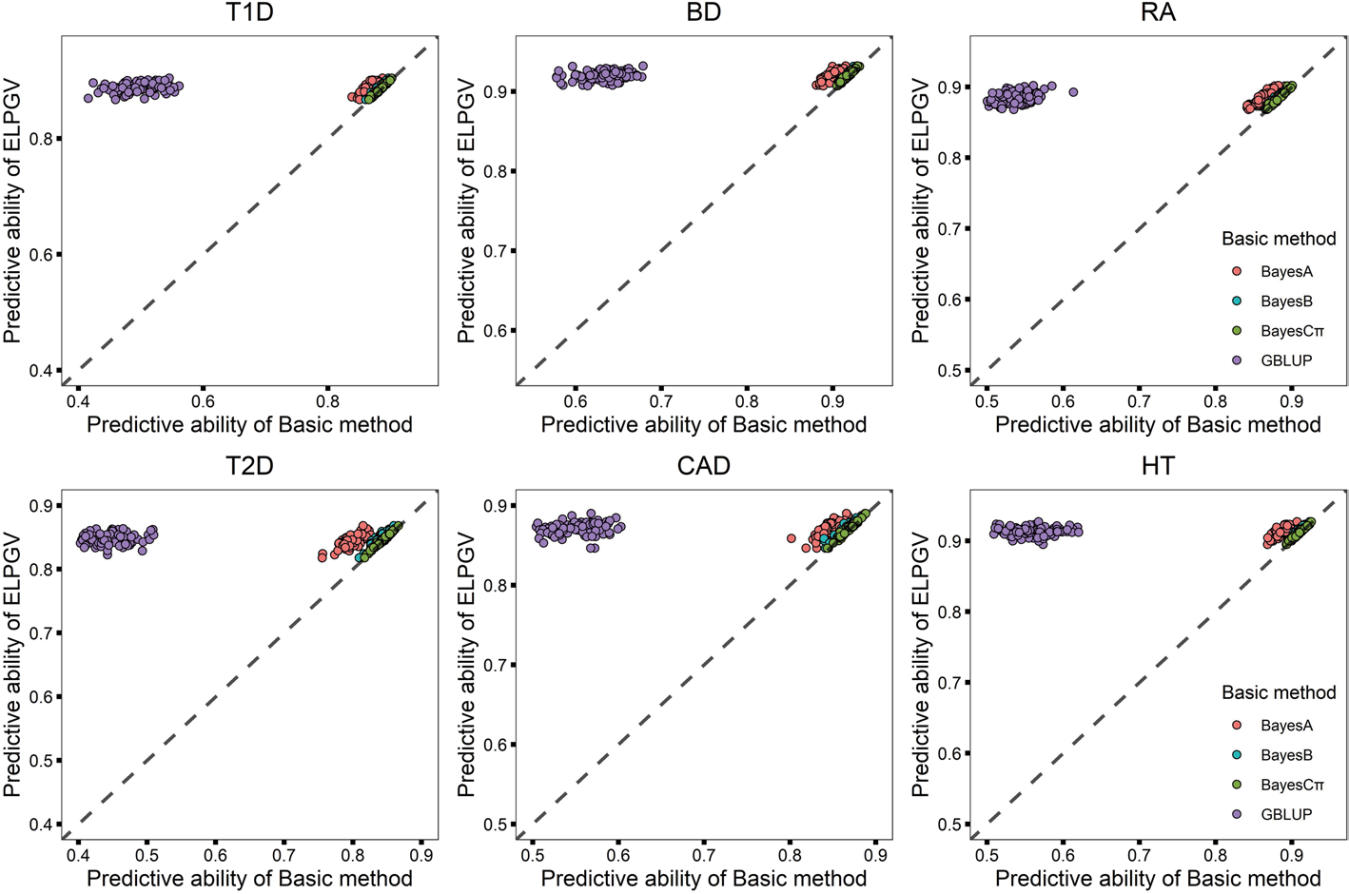
Comparison of the predictive ability of ELPGV and the basic method. **a** T1D, **b** BD, **c** RA, **d** T2D, **e** CAD and **f** HT with WTCCC dataset; different method is denoted with different color, each dot represents single experiment

We first illustrated the results of T2D. Four basic methods, GBLUP, BayesA, BayesB, and BayesCπ were applied for genetic prediction, BayesCπ performed the highest predictive ability (*r* = 0.8471) and GBLUP performed the lowest (*r* = 0.4390) (Table 3). We then used ELPGV to assemble the predictions of four Basic methods to generate new predictions. To this end, we first evaluated the fitting effect of four basic methods with train set, the basic methods with the best fitting effect was used to generate the reference genetic values. The fitting effect was defined as the correlation between the estimated genetic values and the phenotypes in train set. It was found that BayesCπ usually had the best n than other methods. With reference genetic values, ELPGV assembled four basic methods to obtain new predictions, the average predictive ability of ELPGV across 100 validations was *r* = 0.8471, significantly higher than any basic methods with comparison *p*-value ranged from 1.090E−112 (GBLUP) to 6.458E−31 (BayesCπ) Table 3). Because we compared each method with the same dataset, we were able to compare ELGPV with four basic methods in each of 100 experiments, separately. Figure 2a–f shows the prediction abilities in each of experiment, ELPGV is more accurate than other four basic methods, and the advantage of ELPGV over GBLUP is more obvious. We also compared ELPGV with four basic methods in dataset of BD, CAD, T1D, RA and HT (Table 3). For all diseases, ELPGV was obviously more accurate than four basic methods with *p*-values ranged from 4.853E−118 to 9.640E−20 (Table 3).

**Table 3:** The predictive ability of four basic methods and ELPGV, and the comparison *p*-value between ELPGV and others in T1D, T2D, BD, RA, CAD, HT with WTCCC dataset

Method	T1D		T2D		BD		RA		CAD		HT	
	Predictive ability	*p*-value	Predictive ability	*p*-value	Predictive ability	*p*-value	Predictive ability	*p*-value	Predictive ability	*p*-value	Predictive ability	*p*-value
ELPGV	0.8879 ± 0.0008	—	0.8471 ± 0.0009	—	0.9195 ± 0.0006	—	0.8843 ± 0.0008	—	0.8705 ± 0.0008	—	0.9132 ± 0.0006	—
BayesA	0.8664 ± 0.0009	9.037E−69	0.8022 ± 0.0014	9.330E−72	0.8984 ± 0.0007	5.191E−73	0.8628 ± 0.0010	1.454E−68	0.8459 ± 0.0010	6.011E−61	0.8879 ± 0.0007	2.525E−75
BayesB	0.8841 ± 0.0008	4.330E−27	0.8396 ± 0.0010	1.517E−33	0.9167 ± 0.0005	4.416E−32	0.8810 ± 0.0008	1.425E−27	0.8651 ± 0.0008	2.272E−28	0.9100 ± 0.0005	9.319E−29
BayesCπ	0.8863 ± 0.0008	1.827E−23	0.8447 ± 0.0010	6.458E−31	0.9168 ± 0.0006	9.400E−39	0.8827 ± 0.0008	9.640E−20	0.8688 ± 0.0008	1.720E−24	0.9111 ± 0.0006	9.426E−32
GBLUP	0.4957 ± 0.0030	1.597E−114	0.4390 ± 0.0032	1.090E−112	0.6311 ± 0.0023	2.207E−112	0.5337 ± 0.0025	4.853E−118	0.5459 ± 0.0028	7.565E−109	0.5596 ± 0.0028	3.421E−110

ELPGV is the ensemble learning based on BayesA, BayesB, BayesCπ and GBLUP

— Represents no explicit result was found in this method

type 1 diabetes (T1D), type 2 diabetes (T2D), bipolar disorder (BD), rheumatoid arthritis (RA), coronary artery disease (CAD) and hypertension (HT)

### IBD dataset

**Fig. 3:**
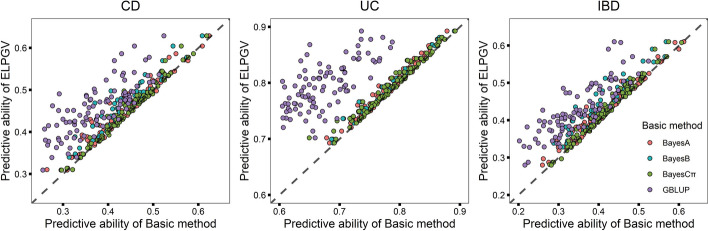
Comparison of the predictive ability of ELPGV and the basic method. **a** CD, **b** UC and **c** IBD with IBDGC dataset; different method is denoted with different color, each dot represents single experiment

We also applied ELPGV to predict disease risk for IBD dataset of European ancestry. The averaged predictive ability of 100 cross-validations of GBLUP, BayesA, BayesB and BayesCπ of UC was 0.6687, 0.7817, 0.7831 and 0.7845, respectively. After assembled with ELPGV, the averaged predictive ability was 0.7920, significantly higher than four basic methods, the *p*-values were from ranged from 3.314E−56 to 3.878E−13 (Table 4). Similarly, the prediction abilities of CD of four basic methods were ranged from 0.3692 (GBLUP) to 0.4452 (BayesCπ), after assembled with ELPGV, the predictive ability was increased to 0.4516, significantly higher than four basic methods (*p*-value varied from 3.659E−34 to 3.938E−07, Table 4). We also show the comparison of each experiment individually, for vast majority of individual experiment, ELPGV outperformed four basic methods, among them, GBLUP performed the lower predictive ability (Fig. 3a–c).

**Table 4:** The predictive ability of four basic methods and ELPGV, and the comparison *p*-value between ELPGV and others in CD, UC, IBD with IBDGC dataset

Method	CD		UC		IBD	
	Predictive ability	*p*-value	Predictive ability	*p*-value	Predictive ability	*p*-value
ELPGV	0.4516 ± 0.0065	—	0.7920 ± 0.0045	—	0.4253 ± 0.0071	—
BayesA	0.4359 ± 0.0067	1.058E−15	0.7817 ± 0.0045	7.365E−25	0.4128 ± 0.0075	2.470 E−13
BayesB	0.4338 ± 0.0063	4.932E−13	0.7831 ± 0.0047	4.845E−16	0.4052 ± 0.0067	1.911E−14
BayesCπ	0.4452 ± 0.0063	3.938E−07	0.7845 ± 0.0047	3.878E−13	0.4171 ± 0.0068	5.272E−10
GBLUP	0.3692 ± 0.0063	3.659E−34	0.6687 ± 0.0052	3.314E−56	0.3455 ± 0.0079	9.965E−35

ELPGV is the ensemble learning based on BayesA, BayesB, BayesCπ and GBLUP

— Represents no explicit result was found in this method

Crohn disease (CD) and ulcerative colitis disease (UC)

### Cattle dataset

**Fig. 4:**
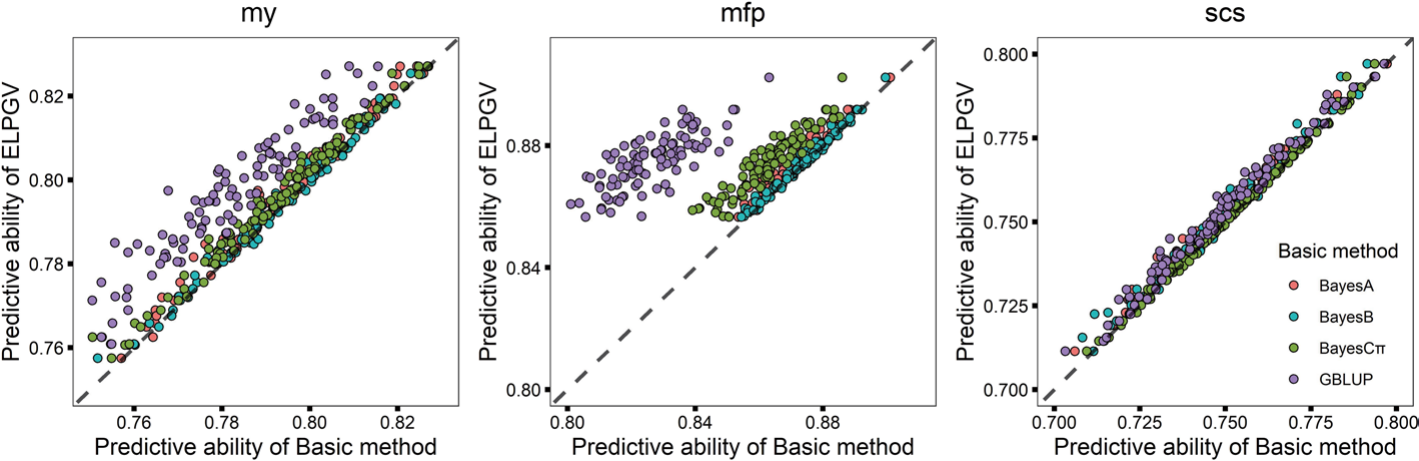
Comparison of the predictive ability of ELPGV and the basic methods. **a** my, **b** mfp and **c** scs with cattle dataset; different method is denoted with different color each dot represents single experiments

We further validated ELPGV with a cattle dataset of German Holstein, in which milk fat percent (mfp), milk yield (my) and somatic cell score (scs) were investigated. For genetic prediction of mfp, BayesCπ performed the highest predictive ability among four basic methods (*r* = 0.8632), whereas GBLUP performed the lowest (*r* = 0.8259) (Table 5). After assembled of four basic methods with ELPGV, the predictive ability was 0.8748, significantly higher than any basic methods (the comparison *p*-values ranged from 9.943E−80 to 2.356E−10, Table 5). The individual experiment showed that for vast majority of the predictions, ELPGV was obviously more accurate than four basic methods, especially than GBLUP (Fig. 4b). For my, ELPGV also outperformed the four basic methods (Fig. 4a) with the comparison *p*-values ranged from 5.133E−52 (GBLUP) to 1.335E−07 (BayesB). For scs, the advantage of ELPGV over four basic methods was also significant, and the *p*-values were ranged from 3.801E−29 (GBLUP) to 0.001 (BayesCπ) (Table 5). Figure 4 shows the accuracies of ELPGV and four basic methods in 100 individual experiments, which displays that for large proportion of predictions, ELPGV has higher prediction abilities than those of four basic methods for all the investigated traits.

**Table 5:** The predictive ability of four basic methods and ELPGV, and the comparison *p*-value between ELPGV and others in mfp, my, scs with cattle dataset

Method	mfp		my		scs	
	Predictive ability	*p*-value	Predictive ability	*p*-value	Predictive ability	*p*-value
ELPGV	0.8748 ± 0.0009	—	0.7959 ± 0.0016	—	0.7523 ± 0.0019	—
BayesA	0.8713 ± 0.0010	2.665E−31	0.7935 ± 0.0017	5.726E−19	0.7496 ± 0.0019	1.242E−23
BayesB	0.8739 ± 0.0009	2.356 E−10	0.7948 ± 0.0017	1.335E−07	0.7503 ± 0.0020	5.614E−11
BayesCπ	0.8632 ± 0.0010	5.884E−52	0.7928 ± 0.0017	1.026E−26	0.7518 ± 0.0019	0.001E−00
GBLUP	0.8259 ± 0.0013	9.943E−80	0.7809 ± 0.0017	5.133E−52	0.7482 ± 0.0019	3.801E−29

ELPGV is the ensemble learning based on BayesA, BayesB, BayesCπ and GBLUP

— Represents no explicit result was found in this method

mfp, milk fat percentage; my, milk yield; scs, somatic cell score

### Wheat dataset

**Fig. 5:**
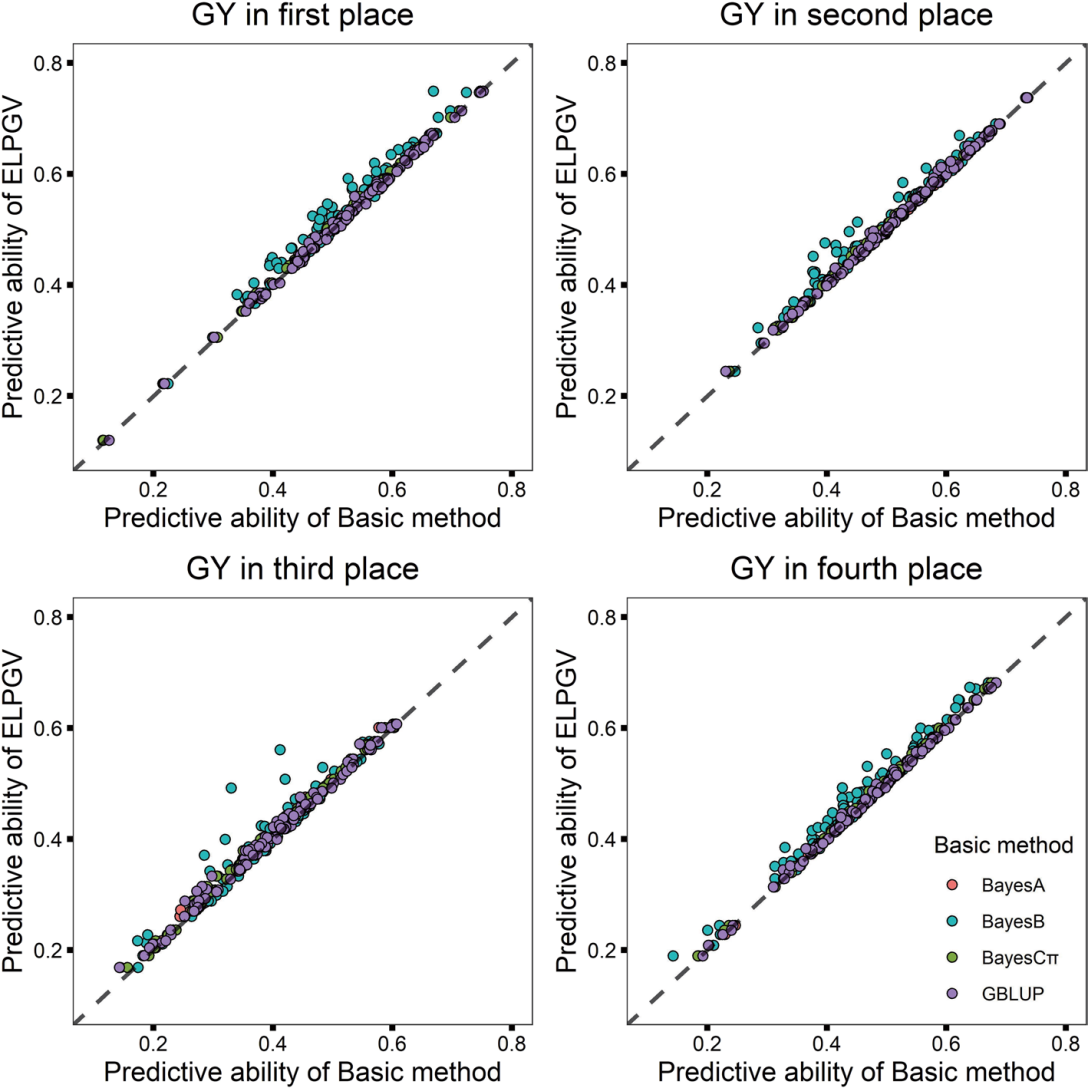
Comparison of the predictive ability of ELPGV and the basic method. **a**–**d** GY (grain yield) under four places of CIMMYT wheat dataset; different method is denoted with different color; each dot represents single experiment

Wheat yields measured under four places were investigated, which includes 599 individuals genotyped for 1279 SNPs. The averaged predictive ability across 100 cross validations of each place is shown in Table 6. For the first place, the prediction abilities of GBLUP, BayesA, BayesB and BayesCπ were 0.5251, 0.5231, 0.5080, and 0.5215, respectively, and the predictive ability of ELPGV was 0.5273, which was significantly higher than four basic methods (the comparison *p*-values ranged from 7.965E−19 to 2.297E−04 (Table 6). For other three places, the results also showed that the prediction accuracy of ELPGV was consistently higher than four basic methods (Table 6). All the predictions of 100 cross-validation are shown in Fig. 5a–d, and ELPGV outperforms four basic methods for majority of single experiments in four places.

**Table 6:** The predictive ability of four basic methods and ELPGV, and the comparison *p*-value between ELPGV and others in GY with wheat dataset

Method	The first place		The second place		The third place		The fourth place	
	Predictive ability	*p*-value	Predictive ability	*p*-value	Predictive ability	*p*-value	Predictive ability	*p*-value
ELPGV	0.5273 ± 0.0104	—	0.5092 ± 0.0101	—	0.4050 ± 0.0102	—	0.4722 ± 0.0101	—
BayesA	0.5231 ± 0.0105	1.550E−10	0.5057 ± 0.0101	1.238E−07	0.3953 ± 0.0103	3.711E−15	0.4676 ± 0.0102	7.265E−14
BayesB	0.5080 ± 0.0104	2.296E−17	0.4947 ± 0.0104	5.993E−12	0.3914 ± 0.0101	4.098E−06	0.4542 ± 0.0101	1.261E−18
BayesCπ	0.5215 ± 0.0105	7.965E−19	0.5046 ± 0.0101	6.695E−11	0.3947 ± 0.0102	2.264E−17	0.4672 ± 0.0102	1.200E−13
GBLUP	0.5251 ± 0.0105	2.297E−04	0.5058 ± 0.0100	7.877E−07	0.3954 ± 0.0104	2.938E−12	0.4698 ± 0.0102	5.013E−05

ELPGV is the ensemble learning based on BayesA, BayesB, BayesCπ and GBLUP

— Represents no explicit result was found in this method

### Simulations

**Fig. 6:**
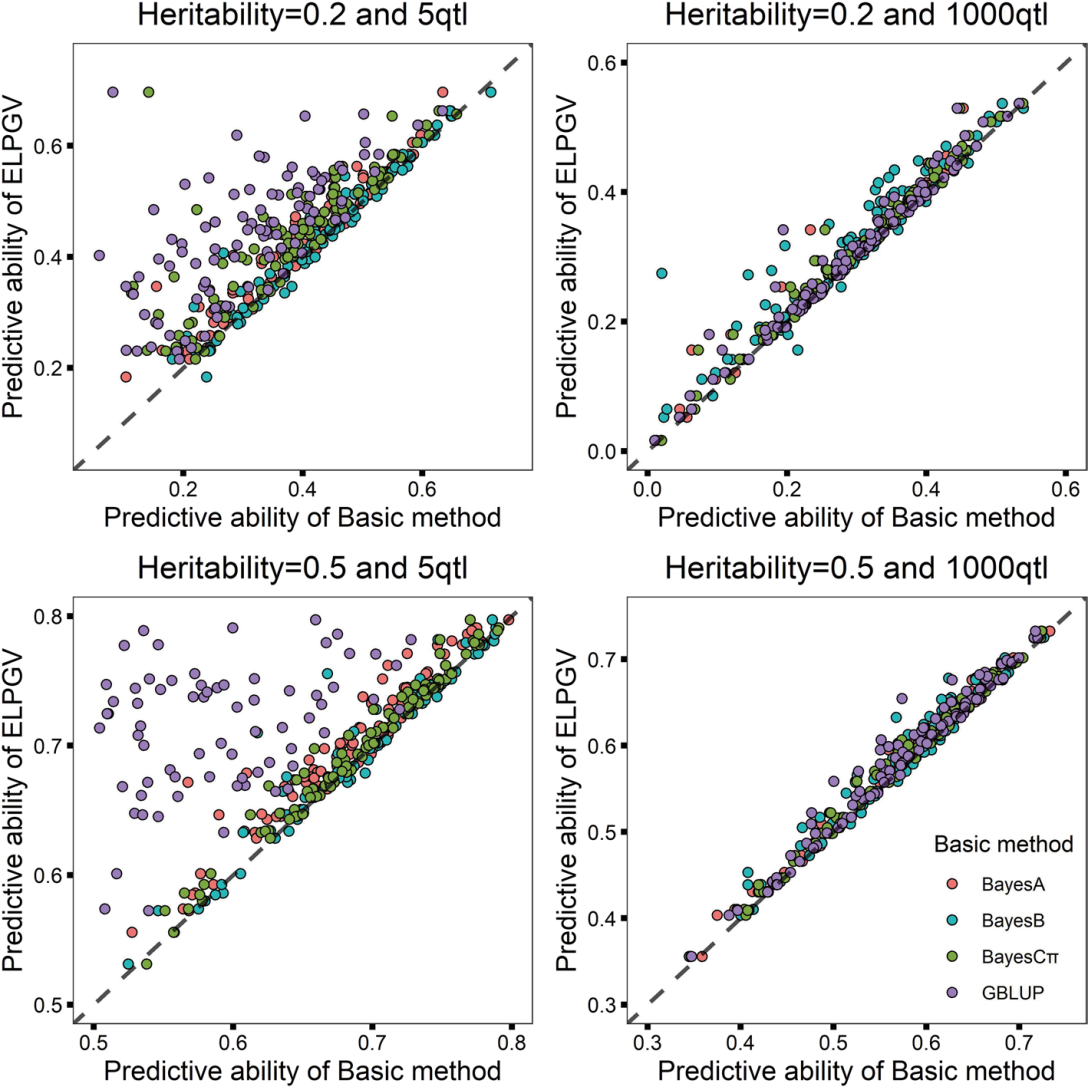
Comparison of the predictive ability of ELPGV and the basic method in simulation. **a** 5 QTL with heritiability 0.2; **b** 1000 QTL with heritiability 0.2; **c** 5 QTL with heritiability 0.5 and **d** 1000 QTL with heritiability 0.5. Different method is denoted with different color, and each dot represents single experiment

We finally performed simulation studies to further investigate the performance of ELPGV. For each group, 100 simulated datasets were generated. Each dataset was randomly divided into 5 parts evenly, and 4 of them were taken as train set and the left 1 part was taken as test set. We first ran four basic methods including GBLUP, BayesA, BayesB and BayesCπ; then assembled the predictions with ELPGV to produce new predictions. For all simulations, ELPGV performed significant higher prediction abilities than corresponding four basic methods, the comparison *p*-values were ranged from 3.553E−34 to 0.001E−00 (Table 7). The 100 replicated experiments also obviously revealed that for each of experiments, the prediction of ELPGV was more accurate than other basic methods (Fig. 6a–d) and the gain of ELPGV over GBLUP was more obvious when QTL number was 5 than 1,000.

**Table 7:** The averaged predictive ability across 100 replications for different methods in 4 scenes of simulation

Method	0.2				0.5			
	5qtl		1000qtl		5qtl		1000qtl	
	Predictive ability	*p*-value	Predictive ability	*p*-value	Predictive ability	*p*-value	Predictive ability	*p*-value
ELPGV	0.4346 ± 0.0116	—	0.3079 ± 0.0109	—	0.7034 ± 0.0064	—	0.5851 ± 0.0085	—
BayesA	0.4042 ± 0.0122	3.317E−15	0.2961 ± 0.0110	1.565E−08	0.6865 ± 0.0068	1.057E−16	0.5765 ± 0.0085	2.652E−17
BayesB	0.4257 ± 0.0118	0.001E−00	0.2842 ± 0.0112	1.096E−08	0.6985 ± 0.0065	0.002E−00	0.5756 ± 0.0086	6.910E−09
BayesCπ	0.3741 ± 0.0140	4.577E−11	0.2956 ± 0.0108	2.828E−10	0.6955 ± 0.0064	1.712E−12	0.5768 ± 0.0086	2.048E−16
GBLUP	0.2952 ± 0.0144	1.032E−22	0.2959 ± 0.0110	6.161E−07	0.5505 ± 0.0096	3.553E−34	0.5727 ± 0.0084	7.160E−13

ELPGV is the ensemble learning based on BayesA, BayesB, BayesCπ and GBLUP

— Represents no explicit result was found in this method

**Fig. 7:**
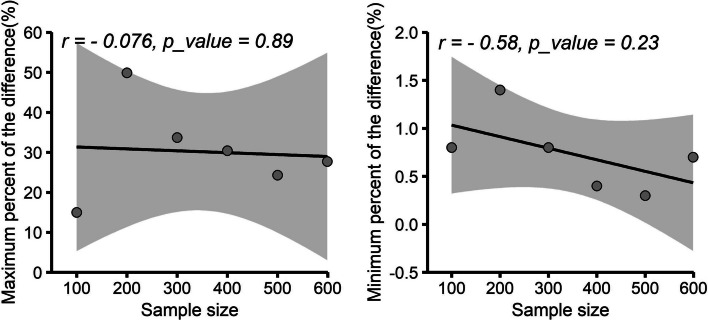
The relationship between sample size and maximum difference or minimum difference of the methods

We next investigated the effect of sample size of training set. We randomly sampled 100, 200, 300, 400, 500 and 599 individuals from wheat data, respectively, the QTL number was set as 5 and the heritability was 0.5. For each of sample sizes, 100 independent datasets were generated. The cross validation was used to evaluate the prediction abilities. It revealed that the prediction abilities of ELPGV were higher than four basic methods for all simulated sample size (Table 8). We next investigated if the advantage of ELPGV over other methods was dependent on the sample size. To do this, we summarized the maximum and minimum difference of the prediction abilities between ELPGV and other methods, respectively (Table 8) and correlated the maximum (Fig. 7a) or minimum (Fig. 7b) differences to the corresponding sample sizes. But we did not find evidence of significant correlation (*r* = − 0.58 and − 0.076 with *p*-value 0.23 and 0.89), which implies that the gain of ELPGV over basic methods is not affected by sample size.

**Table 8:** The maximum and minimum difference of the predictive ability between ELPGV and other methods in different sample size of simulation

Method	100	200	300	400	500	599
	Predictive ability	Predictive ability	Predictive ability	Predictive ability	Predictive ability	Predictive ability
ELPGV	0.6055 ± 0.0220	0.7175 ± 0.0159	0.7559 ± 0.0085	0.7189 ± 0.0070	0.7378 ± 0.0061	0.7034 ± 0.0064
BayesA	0.5486 ± 0.0256	0.6827 ± 0.0170	0.7200 ± 0.0085	0.7034 ± 0.0073	0.7231 ± 0.0069	0.6865 ± 0.0068
BayesB	0.6007 ± 0.0237	0.7075 ± 0.0170	0.7502 ± 0.0084	0.7158 ± 0.0069	0.7344 ± 0.0060	0.6985 ± 0.0065
BayesCπ	0.5334 ± 0.0215	0.6215 ± 0.0266	0.7271 ± 0.0100	0.7069 ± 0.0076	0.7354 ± 0.0060	0.6955 ± 0.0064
GBLUP	0.5266 ± 0.0216	0.4785 ± 0.0243	0.5655 ± 0.0151	0.5511 ± 0.0143	0.5934 ± 0.0095	0.5505 ± 0.0096
Maximum difference	0.0789 (15.0%)	0.2390 (49.9%)	0.1904 (33.7%)	0.1678 (30.4%)	0.1444 (24.3%)	0.1529 (27.7%)
Minimum difference	0.0048 (0.8%)	0.0100 (1.4%)	0.0057 (0.8%)	0.0031 (0.4%)	0.0024 (0.3%)	0.0049 (0.7%)

ELPGV is the ensemble learning based on BayesA, BayesB, BayesCπ and GBLUP

## Discussion

We have presented an ensemble learning method, ELPGV to predict genetic values. The key feature of ELPGV is that it assembles predictions of other basic methods into more accurate predictions. Extensive datasets of human, cattle and wheat have been employed to validate the performance of ELPGV, all results consistently revealed that ELPGV was able to integrate the merit of each method together to produce significantly higher predictive ability than any basic methods. Based on these advantages, ELPGV is expected to be widely used for prediction in large data sets.

Ensemble learning has been widely utilized in genome selection, such as Ma et al*.* [36], who assembles two basic methods and trains the weights with PSO algorithm; however, it has several disadvantages, (1) it assumes the phenotypes of testing individuals have been known, so that it is only applicable for prediction with known phenotype, which is less meaningful in practice; (2) the performance of the traditional PSO greatly depends on its parameters, and it often suffers from being trapped in local optima [37, 38], which is consistent with the study of Cai et al*.* [39]. Liang et al*.* [40] construct a stacking ensemble learning framework (SELF), integrating three machine learning methods and an ordinary least square regression was chosen as the meta learner, to improve the genomic predictions. A lot of experiment indicated that SELF with the great potential to improve genomic predictions in other animal and plant populations. In actual analysis, SELF taken the genomic relationship matrix derived by genotypes as the inputs directly. But this might reduce the prediction accuracy of a single basic method. Additionally, Gianola et al. [41] was found that bagging can ameliorate predictive performance of GBLUP and make it more robust against over-fitting. However, because of predictive ability increases with training set size [42]. It is obvious that bagging may not be feasible for immense data sets.

DE algorithm is another kind of evolutionary algorithm, which has been applied to a series of problems arising in various fields of science, engineering, and management [43–45]. In our analysis, we found that DE algorithm is much more stable, and always converge to the same solution after repeated operations; furthermore, DE converges fast and is very accurate for high-dimensional problems, which has three main parameters (initialize solution size, scaling factor $F$, crossover probability $CR$), but it is not sensitive for parameter setups [46]. While DE algorithm has many advantages, the disadvantage of it is that it is difficult to update model parameters [46], but PSO does not have this problem. So, hybridization is an important modification in DE which is implemented to enhance its performance and convergence speed. Plenty of work can be found in the literature on the hybridization of DE. For instance, Pant et al. [47] proposed a hybrid version of DE with PSO and results show that the proposed DE-PSO is quite competent for solving the considered test functions as well as real-life problems. Zhang et al. [48] proposed a hybrid technique using DE with PSO for unconstrained optimization problems. Similarly, ELPGV is the hybrid of DE and PSO too, which not only inherits the high precision merit of DE algorithm, but also possesses the fast convergence character of PSO algorithm.

In the prediction of the disease risk for human, ELPGV exhibits greater advantages over four basic methods. In almost all of situations, ELPGV is more accurate than others, the gain is much more obvious when comparing with GBLUP, reflecting that GBLUP is not very suitable for human dataset, may be due to the fact that the relationships between individuals are quite limited and few information is available for GBLUP predictions. In contrast, the situation is quite different for cattle and wheat datasets. The reason may be that the aim of these datasets is for selection breeding and the individuals have extensive relationship, which is consistent with the literature [49]. Additionally, Heslot et al. [50], Azodi et al. [51] and Schrauf et al. [52] also compared GBLUP (or equivalent models) with other genomic prediction methods in a variety of plant datasets and have shown that the difference between GBLUP and other methods is negligible under large data sizes and polygenic architectures. Because the GBLUP efficiently predicts individual genetic values using the relationship information, and all markers are assumed in a sense to contribute equally to the construction of Kinship matrix.

It is shown that the performance of ELPGV is greatly affected by the method similarity, which is consistent with Granitto et al. [53] who concludes diverse basic methods is an essential characteristic of a good ensemble method. Therefore, one way to improve the performance of ELPGV is to increase the diversity of basic methods. For example, BayesB, BayesCπ and BayesR [54] are working well for major-effect QTL method, they often performed similar prediction abilities, so integrating them would not enhance the predictive ability of ELPGV too much; similarly, rrBLUP [55] is theoretically quite similar to GBLUP, both are based on polygenic method, it would not substantially increase the predictive ability by integrating them together.

We have proposed ELPGV method for optimizing the parameters, which greatly improves the precise of parameter estimates. It's versatility to allow for different and more complex criterion to be maximized. However, it still has room to improve, for example, combining DE or PSO with other optimization algorithms to form a better hybrid algorithm [46], or using other ensemble strategies, such as sequence integration methods such as boosting method [56].

## Conclusions

We have presented an ensemble learning method, ELPGV, to predict genetic values. The key feature of ELPGV is that it assembles predictions of other basic methods into more accurate predictions. ELPGV is able to integrate the merit of each method together to produce significantly higher predictive ability than any basic methods and it is simple to implement, which uses only the predictions of basic methods as input without using genotype data. Therefore, ELPGV requires quite few computers RAM and can complete task even with PC computer; furthermore, ELPGV is computationally fast, which takes only several minutes to complete the assembling for tens thousands of individuals and is promising for wide application in genetic predictions.

## Data Availability

The ELPGV scripts are available in the release package on github (https://github.com/GuLinLin/ELPGV). The detailed user manual is available from https://github.com/GuLinLin/ELPGV/blob/main/ELPGV_User_Manual.pdf. This study makes use of data generated by the Wellcome Trust Case Control Consortium (WTCCC). A full list of the investigators who contributed to the generation of the WTCCC data is available from http://www.wtccc.org.uk/, where the dataset can be publicly accessed. Funding for the WTCCC project was provided by the Wellcome Trust under award 076113 and 085475. The following datasets can be directly downloaded from the links below: Cattle: https://academic.oup.com/g3journal/article/5/4/615/6025251. Wheat: http://cran.r-project.org/web/packages/BLR/index.html. The IBD dataset of this study are available from the international IBD Genetics Consortium (https://www.ibdgeneticsa.org/) by reasonable application.
